# Maplirpacept: a CD47 decoy receptor with minimal red blood cell binding and robust anti-tumor efficacy

**DOI:** 10.3389/fimmu.2025.1518787

**Published:** 2025-02-26

**Authors:** Mithunah Krishnamoorthy, Ruth Seelige, Christopher R. Brown, Nancy Chau, Natasja Nielsen Viller, Lisa D. S. Johnson, Emma Linderoth, Jean C. Y. Wang, Christopher P. Dillon, Keith Abayasiriwardana, Clare Lees, Mark Wong, Megan M. Kaneda, Robert A. Uger, Gloria H. Y. Lin

**Affiliations:** ^1^ Pfizer Oncology, Pfizer Inc., La Jolla, CA, United States; ^2^ Research and Development, Trillium Therapeutics Inc., Mississauga, ON, Canada; ^3^ Princess Margaret Cancer Centre, University Health Network, Toronto, ON, Canada; ^4^ Division of Medical Oncology and Hematology, Department of Medicine, University Health Network, Toronto, ON, Canada; ^5^ Department of Medicine, University of Toronto, Toronto, ON, Canada

**Keywords:** CD47, phagocytosis, red blood cells, hematological malignances, SIRP alpha

## Abstract

**Introduction:**

CD47 is highly expressed on cancer cells and triggers an anti-phagocytic “don’t eat me” signal when bound by the inhibitory signal regulatory protein α (SIRPα) expressed on macrophages. While CD47 blockade can mitigate tumor growth, many CD47 blockers also bind to red blood cells (RBCs), leading to anemia. Maplirpacept (TTI-622, PF-07901801) is a CD47 blocking fusion protein consisting of a human SIRPα fused to an IgG4 Fc region and designed to limit binding to RBCs.

**Methods:**

To determine maplirpacept binding to RBCs and interference with blood tests, human blood samples were used. The ability of maplirpacept to promote macrophage-mediated phagocytosis of human tumor cells was assessed using both confocal microscopy and flow cytometry. *In vivo* antitumor efficacy as a monotherapy and in combination with other therapeutic agents was evaluated in xenograft models.

**Results:**

In the current study, we demonstrate that maplirpacept has limited binding to RBCs while driving enhanced macrophage-mediated phagocytosis of hematological tumor cells *in vitro* and reducing tumor burden in human xenograft models. Moreover, phagocytosis of neoplastic cells can be enhanced when maplirpacept is combined with other therapeutic agents, including antibodies or chemotherapeutic agents.

**Conclusion:**

These preclinical results establish maplirpacept as an effective CD47 blocker that mitigates the potential for anemia in patients.

## Introduction

CD47 is a ubiquitously expressed transmembrane glycoprotein that binds the signal regulatory protein alpha (SIRPα) on macrophages. This binding delivers an inhibitory (“don’t eat me”) signal to macrophages, suppressing phagocytosis. CD47 is overexpressed in many tumors and is associated with poor overall survival (1), suggesting that tumor cells have evolved to upregulate the expression of CD47 to escape immune mediated clearance. For example, malignant cells from patients with B-cell non-Hodgkin lymphoma (B-NHL), including diffuse large B cell lymphoma, (DLBCL), mantle cell lymphoma, (MCL), follicular lymphoma (FL), and marginal zone lymphoma (MZL), exhibited elevated CD47 expression (average 2-fold) compared to normal B cells in the periphery and in germinal centers (2). Additionally, the increase in CD47 expression correlated with increased disease severity in the case of low–risk myelodysplastic syndrome (MDS) to high-risk MDS and the subsequent transition to acute myeloid leukemia (AML) (3). Elevated CD47 expression was also observed on disseminated B-NHLs cells compared to primary lesions in a preclinical mouse model (4). Thus, blocking the CD47-SIRPα axis has emerged as a novel therapeutic strategy for hematological malignancies and has demonstrated preclinical efficacy *in vitro* and *in vivo* (5, 6).

While CD47 is highly expressed on neoplastic cells, it is also expressed on RBCs, presenting several risks for potential CD47 blocking agents (7). Preclinical studies have shown that while anti-CD47 antibodies can trigger increased phagocytosis of tumor cells, they can also induce phagocytosis of red blood cells (RBCs), eliciting phagocytic anemia (8, 9). Moreover, CD47 expression on RBCs presents a large antigen sink that could hinder the dissemination of CD47 blocking agents to tumors. Therefore, design of novel CD47 blockers should aim to mitigate these risks in addition to facilitating phagocytosis of tumor cells.

Maplirpacept (TTI-622, PF-07901801) is a CD47 blocking fusion protein designed to limit binding to RBCs. This construct consists of two wildtype CD47 binding domains of human SIRPα fused to a human IgG4 Fc domain, which provides a moderate strength pro-phagocytic signal through interactions with Fcγ receptors. Human SIRPα has been reported to have moderate CD47 binding affinity (10) compared to anti-CD47 mAbs (11, 12), therefore we hypothesized that this may reduce binding to RBCs. In the current study, we demonstrate that maplirpacept also has limited binding to RBCs; potentially reducing the risk for phagocytic anemia that is associated with anti-CD47 mAb approaches. This minimal binding potentially precludes maplirpacept from interfering with routine blood tests. Despite the moderate binding affinity to human CD47, maplirpacept drives enhanced macrophage-mediated phagocytosis of a wide variety of hematological tumor cells *in vitro* and reduces tumor burden in human xenograft models. Collectively, these studies demonstrate that maplirpacept facilitates enhanced phagocytosis of malignant cells, especially when combined with other therapeutic agents, including antibodies or chemotherapeutic agents. Thus, these preclinical results establish maplirpacept as an attractive CD47 blocker that mitigates the potential for anemia in patients (13).

## Materials and methods

### SIRPαFc Proteins

Maplirpacept consists of the N-terminal V domain of human SIRPα (GenBank AAH26692) fused to the human IgG4 Fc region (hinge-CH2-CH3, UniProtKB/Swiss-Prot, P01861). This fusion protein contains a hinge-stabilizing mutation that prevents the formation of intrachain disulfide bonds (14). All constructs were generated by overlapping PCR using standard molecular biology techniques and expressed in stably transfected CHO-S cells (Invitrogen). Proteins were purified from cell culture supernatant using protein A and hydrophobic interaction chromatography, concentrated, and residual endotoxin removed. Control human IgG4 lacking the SIRPα domain (Fc control) were also generated and similarly purified. All proteins displayed >99% purity by HPLC and <0.1 EU/mg endotoxin.

### Cells

The following cell lines were used: DLBCL (DOHH2 (RRID: CVCL_1179), NUDUL1 (RRID: CVCL_1877), Pfeiffer (RRID: CVCL_3326), SU-DHL-6 (RRID: CVCL_2206), SU-DHL-8 (RRID: CVCL_2207), SU-DHL-16 (DSMZ Cat# ACC-577, RRID: CVCL_1890), Toledo (RRID: CVCL_3611)), acute myeloid leukemia (MV-4-11 (RRID: CVCL_C3HG), OCI-AML2 (RRID: CVCL_1619)), non-DLBCL lymphomas (Raji (RRID: CVCL_0511), Daudi (RRID: CVCL_0008)), multiple myeloma (8226(RRID: CVCL_0014), H929 (RRID: CVCL_1600), MM1.S (RRID: CVCL_8792), MOLP8-luc (RRID: CVCL_2124)), T cell lymphoma (Karpas 299 (RRID: CVCL_1324)). All cell lines were obtained from ATCC except DOHH2 (DSMZ), MOLP-8 (DSMZ), OCI-AML2 (DSMZ) and Karpas 299 (Sigma-Aldrich).

Frozen primary tumor cells from the peripheral blood or bone marrow of patients with B-cell acute lymphoblastic leukemia (ALL), T- ALL, MDS, and AML were obtained from the University Health Network (UHN) BioBank (Toronto, Canada) according to the procedures approved by the Research Ethics Board of UHN.

Human macrophages were differentiated from monocytes either purchased frozen (Stemcell, Charles River) or separated from peripheral blood mononuclear cells (PBMCs) from healthy donors (BioIVT) via CD14^+^ negative selection kit (StemCell). Monocytes were differentiated into macrophages by culturing for at least 7 days in X-Vivo-15 media (Lonza) supplemented with 20 ng/mL M-CSF (PeproTech). 24 hours prior to phagocytosis assays, the monocyte-derived macrophages were primed with 300 ng/mL IFNγ (PeproTech, R&D) to generate M1 macrophages or with 10 ng/mL IL-10 (R&D) to generate M2 macrophages. When required, macrophages were harvested using Enzyme-Free Cell Dissociation Buffer (ThermoFisher) or TrypLE (Gibco). M1 and M2 macrophages polarization were confirmed to be MHCII^+^ or CD206^+^ respectively via flow cytometry.

For phagocytosis assays conducted with non-malignant cells: non-malignant cells were isolated from the whole blood of healthy human donors (Biological Specialty Corp). Platelets and RBCs were isolated from plasma by differential centrifugation. T cells, B cells and monocytes were isolated directly from whole blood using antibody coated magnetic beads (Stemcell RosetteSep). Platelets were isolated from plasma by differential centrifugation.

### Tumor cell binding

Cell lines or primary patient samples were incubated with titrated amounts of biotinylated maplirpacept or biotinylated isotype-matched control IgG Fc, together with Near-IR LIVE/DEAD Fixable Dead Cell Stain (Invitrogen) for 30 minutes on ice. Cells were washed, stained with phycoerythrin (PE)-conjugated streptavidin (eBioscience), washed, and resuspended in Stabilizing Fixative (BD Biosciences). Flow cytometry was performed on a FACSVerse flow cytometer (BD Biosciences). Data were analyzed using FlowJo software (BD Biosciences). Half-maximal effective concentration (EC_50_) values were calculated using a sigmoidal dose–response curve in GraphPad Prism software.

### Red blood cell binding

RBCs were isolated from sodium-heparinized whole blood from healthy human donors (Biological Specialty Corporation) by centrifugation followed by several washes with PBS. The resulting packed RBCs were diluted in PBS. Binding was performed by incubating RBCs with titrated amounts of maplirpacept, anti-CD47 mAbs [BRIC126 (Serotec), 2D3 (eBioscience), CC2C6 (BioLegend), B6H12 (in-house), hu5F9 (in-house)]. Cells were washed and subsequently stained with biotin-conjugated anti-human IgG Fc PAN (Hybridoma Reagent Laboratory), followed by detection with PE-conjugated streptavidin (eBioscience). Flow cytometry was performed on a FACSVerse flow cytometer (BD Biosciences).

### Hemagglutination assays

Titrated amounts of maplirpacept or anti-CD47 mAbs (up to 3 μM) were added to wells containing RBCs diluted in PBS, were incubated overnight at 37°C in 5% CO2. The extent of hemagglutination was assessed by scoring each well on a scale of 1 to 6, with 1 representing the absence of hemagglutination and 6 representing complete hemagglutination.

### Blood testing

Test samples were prepared using whole blood from healthy donors representing all 8 ABO-Rh blood groups collected into EDTA (Vacutainer EDTA tubes, Becton Dickinson) with maplirpacept or anti-CD47 mAb (Hu5F9) spiked in at indicated concentrations. Whole blood was then diluted to 5% with sterile saline before subsequent addition to Anti-A, Anti-B, Anti-AB or Anti-D immunoglobulins in a 1:1 v/v ratio. Samples were then centrifuged before being agitated to dislodge the RBC pellet. The presence (+) or absence (-) of agglutination was determined by two independent blinded reviewers.

### Indirect anti-globulin test (cross matching)

Blood from healthy donors was collected into EDTA (Vacutainer EDTA tubes, BD). Five percent RBC solutions were generated by adding whole blood to sterile saline and plasma was collected from whole blood via centrifugation. Test samples were generated by spiking plasma with maplirpacept or anti-CD47 mAb (clone: Hu5F9, Selleckchem) at indicated concentrations.

5% RBC solutions were mixed with plasma test samples in 1:2 v/v ratio and incubated at 37°C for 30 to 60 min. RBCs were then pelleted via centrifugation and washed with saline before resuspension with anti-human Anti-IgG. Plates were centrifuged at 1000 x g for 20 seconds and agitated to dislodge the pellet. The presence (+) or absence (-) of agglutination was determined by two independent blinded reviewers (without the aid of a microscope).

### Confocal-based phagocytosis assay

Non -malignant cells, tumor cells and/or platelets were labeled with either CellTrace CFSE (Life Technologies) or violet proliferation dye 450 (BD Biosciences) and added to polarized macrophages at an effector:target (E:T) ratio of 1:5 or 1:40 for assays containing tumor cells or platelets respectively. M1 polarized macrophages and tumor cells or platelets were cocultured for 2 hours at 37°C in 5% CO2 in the presence of maplirpacept or Fc control protein and subsequently stained with Alexa Fluor 555–conjugated Wheat Germ Agglutinin (Invitrogen). Phagocytosis was assessed by confocal microscopy on a Quorum Wave FX-X1 Spinning Disc Confocal System and images were analyzed using Volocity software (Quorum Technologies). A phagocytosis index was calculated as: (number of non malignant cells, tumor cells, or platelets inside macrophages/number of macrophages) × 100; counting at least 200 macrophages per sample. All tumor or platelets cells counted were confirmed to be internalized using z-stack images. Statistical significance was calculated by unpaired t test versus isotype control, one-way ANOVA or two-way ANOVA using GraphPad Prism software.

### Flow cytometry–based phagocytosis assay

Tumor cells (labeled with either Violet Proliferation Dye 450 [BD Biosciences] or CellTrace CFSE [Life Technologies]) were co-cultured with polarized macrophages at either a 1:2 or 1:5 E:T ratio for 2 hours at 37°C, in the presence of maplirpacept or control Fc protein. Macrophages were labeled with CellTrace Violet (Life Technologies) pre incubation or APC-conjugated anti-human CD14 (61D3, eBioscience), and PE-conjugated anti-human CD11b (ICRF44, eBioscience) post incubation. All samples were stained with Near-IR LIVE/DEAD Fixable Dead Cell Stain (Invitrogen) prior to sample acquisition. Samples were acquired on a FACSVerse, BD fortessa or BD Canto flow cytometer, and data were analyzed using FlowJo software (Treestar Inc.). Percent phagocytosis was reported as the percent of live macrophages that were positive for tumor cell label. M1 polarized macrophages were utilized unless stated otherwise. Statistical significance was calculated by unpaired t-test or one-way ANOVA using GraphPad Prism software.

### Viability assays

Tumor cell line and macrophage viability were determined using the Cell Titer Fluor assay (Promega) according to manufacturer’s protocol. Briefly, 1000X GF-AFC substrate was diluted to 1X in assay buffer and subsequently added at a ratio of 1:1 to cell suspension (5 x 10^4^ tumor cells or MDMs/well). Cells and diluted GF-AFC were incubated at 37°C for at minimum 30 min before fluorescent signal was quantified using a Spectramax i3 (excitation/emission 400nm/505nm). Percent viability was calculated by subtracting background fluorescence and normalizing values as a percent of DMSO only control (see equation below).


$$\%\text{viability}\;\text{of}\;\text{sample}=\frac{sample\;-\;media\;only\;}{DMSO\;only\;-\;media\;only}\;\times 100$$

### Subcutaneous xenograft models

NOD.SCID (NOD.Cg-Prkdc^scid^/J), CB17. SCID (C.B-*Igh-1^b^*/IcrTac-*Prkdc^scid^*) or NSG (NOD.Cg-Prkdc^scid^ Il2rg^tm1Wjl^/SzJ) mice were implanted subcutaneously with tumor cell lines in 50% Matrigel as indicated by **Supplementary Table 1** below. In certain models, mice were irradiated prior to implantation (see **Supplementary Table 1**). Treatments were initiated when tumor volumes averaged ~100-150mm^3^ in volume. Mice were dosed as indicated in **Supplementary Table 1**. Tumor volumes were estimated twice weekly by standard caliper measurements of length and width then calculated as follows: 0.5 × (longest diameter[mm]) × (shortest diameter [mm])^2^. Tumors were measured until maximum permissible volume (1500-2250 mm^3^, depending on model). If clinical signs or >20% body weight loss occurred, mice were euthanized. All procedures performed on animals were in accordance with regulations and established guidelines that were reviewed and approved by an institutional animal care and use committee.

### Intravenous xenograft model

NSG-MHC I/II DKO (NOD.Cg-Prkdc^scid^ H2-K1^b-tm1Bpe^ H2-Ab1^g7-em1Mvw^ H2-D1^b-tm1Bpe^ Il2rg^tm1Wjl^/SzJ) mice purchased from Jackson Laboratories were irradiated with 100 cGy then implanted with 2 x 10^6^ luciferase expressing MOLP-8 cells intravenously. Tumor growth was monitored 1–2 times every week by bioluminescent imaging (BLI). Briefly, mice were injected intraperitoneally with 3 mg of luciferin-D (Promega) 15 minutes prior to BLI acquisition (IVIS Imaging System, Perkin Elmer, Waltham, Massachusetts) under isoflurane anesthesia. The results of BLI were reported as total photon flux/second (p/s). Dosing of maplirpacept (10 mg/kg, SC,QW) was initiated when BLI reached 3.5x 10^7^ p/s. All procedures performed on animals were in accordance with regulations and established guidelines that were reviewed and approved by an institutional animal care and use committee.

### Primary AML xenograft models

AML patient xenograft models were established in 10-week-old female NOD.SCID (NOD.Cg-Prkdc^scid^/J) mice bred and maintained in the Barrier Unit at the UHN Animal Facility (Toronto, Canada). One day prior to transplantation, mice were sublethally irradiated (275 cGy) and pretreated with anti-CD122 antibody (0.2 mg/mouse) to deplete residual host NK cells. On the day of transplantation, viably frozen mononuclear cells collected from a single AML patient were thawed, counted, and transplanted intrafemorally into the preconditioned mice at a dose of 5 × 10^6^ cells/mouse. Two weeks after transplantation, mice received the indicated doses of maplirpacept or control human IgG1 Fc 3 times/week for 4 weeks. At study termination, bone marrow from injected and non-injected femurs was collected and stained with mouse anti-human antibodies including CD47-FITC, CD33-PE, CD19-PE-Cy5, CD45-APC, CD34-APC-Cy7, CD38-PE-Cy7. After staining, washed cells were run on an LSRII flow cytometer (BD Biosciences). Collected data were analyzed by FlowJo software to assess AML engraftment levels in the injected femur and noninjected bones as determined by the percentage of human CD45^+^CD33^+^ cells. All procedures performed on animals were in accordance with regulations and established guidelines that were reviewed and approved by an institutional animal care and use committee.

### Statistical analysis

Under the assumption of independent variables, normal distribution and equal variance of samples, statistical significance was assessed using unpaired t-tests, one-way ANOVA or two-way ANOVA for indicated *in vitro*, *ex vivo* and primary AML xenograft *in vivo* experiments.

For *in vivo* studies in which tumor growth over time was assessed (all studies except primary AML xenograft study), tumor growth inhibition (TGI) is reported as delta TGI that accounts for tumor size at randomization and was calculated when the first mouse in the control group reached tumor volume endpoint, based on the equation:


$$TGI\%\left(1-\frac{V_{T}-V_{TO}}{V_{C}-V_{CO}}\right)\times 100\%$$

where is the average volume of treatment group at time T , is the average volume of treatment group at Day 0, is the average volume of control group at time T , is the average volume of control group at Day 0.

Statistical significance for TGI was determined by one-way (for comparisons against control group) or two-way (for all other comparisons) ANCOVA from T/C values, where


$$T/C=\left(1-\frac{V_{T}}{V_{C}}\right)\times 100$$

Testing for additivity vs. synergy for combinatorial treatments was done with the Bliss equation: $S=\;\log\left(V_{12}\right)+\;\log\left(V_{0}\right)-\log\left(V_{1}\right)-\;\log\left(V_{2}\right)$ , where S<0 indicates synergy. S was calculated as equal to the average log volumes of the combination ( $V_{12}$ ) plus average log volumes of control ( $V_{0}$ ) minus the average log volumes of the single therapies ( $V_{1}$ and $V_{2}$ ). S^σ, with σ being the residual standard deviation, is shown to follow a t -distribution with $N_{12}+N_{0}+N_{1}+N_{2}-4$ degrees of freedom ( $N_{j}$ is the number of animals assigned to group j ) and tested for the null hypothesis of $H_{o}:S=0\;$ with alternative hypothesis of $H_{1}:S<0$ (one-sided since we are only looking for synergy not antagonism). If the p-value of the combinatorial treatment was p <0.05 compared to Bliss, synergistic rather than additive activity for the combination treatment was determined.

Statistical significance for survival was determined by Log-rank (Mantel-Cox) test. Error bars presented in figures indicate mean ± SEM. Statistical tests were performed using GraphPad Prism v9.5.1 or Pfizer-internal software.

### Western blotting

Indicated protein amounts were diluted in LDS sample buffer with (reducing) or without (non-reducing) beta –mercaptoethanol, were resolved by 4% -12% Bis-Tris –polyacrylamide gel electrophoresis and analyzed by Western blotting with antibodies against anti-hIgGFc (goat anti-hIgG fc, Sigma Cat # I1886; Rabbit anti goat IgG HRP, Sigma Cat #A5420).

## Results

### Maplirpacept binds minimally to RBCs and does not interfere with blood transfusion compatibility tests

**Figure 1:**
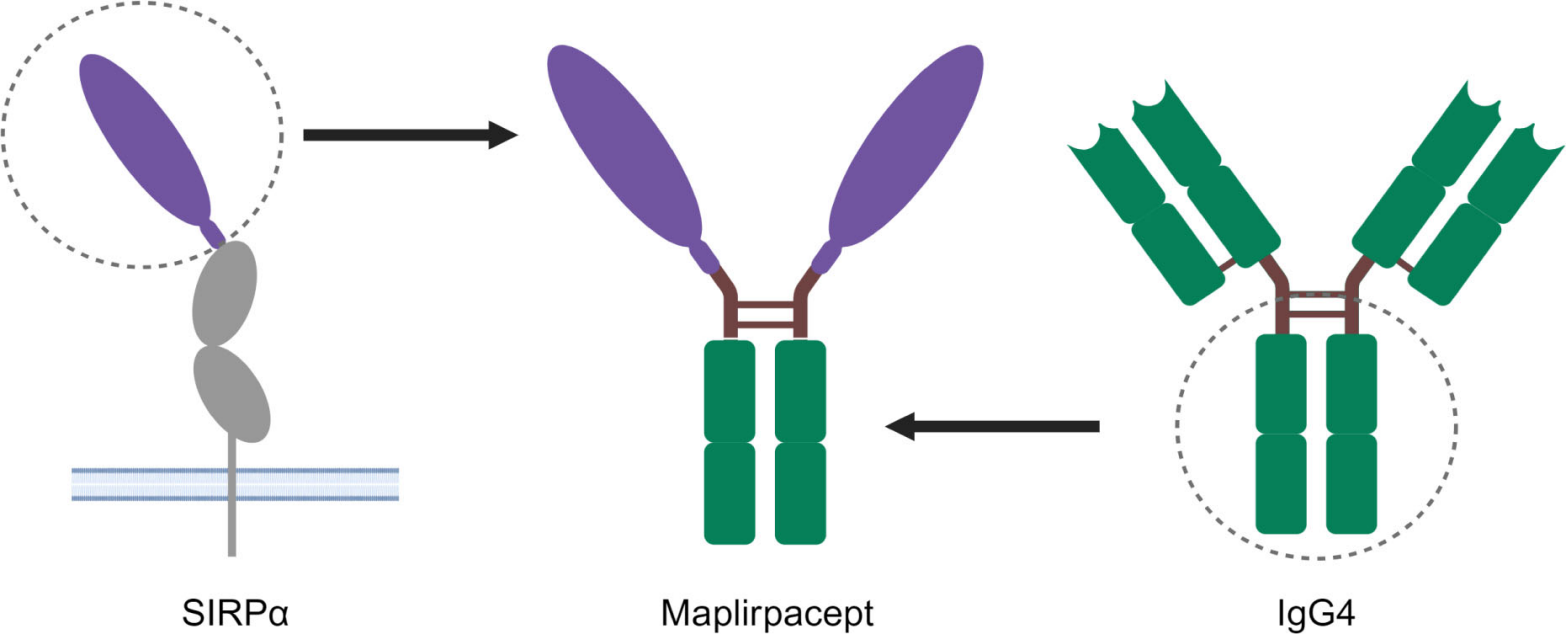
Structure of maplirpacept. Maplirpacept consists of the N-Terminal domain of human SIRPα (shown in purple) linked to a human IgG4 Fc Region (shown in green). The hinge and interchain disulfide bonds are shown as brown lines. Created with BioRender.com

CD47 is expressed on all cells, including RBCs, where loss of CD47 expression is important for the clearance of aging RBCs (7). Previous generations of CD47 blockers such as anti-CD47 mAbs bind to CD47 on RBCs and elicit anemia (8). To mitigate the binding of RBCs observed with other CD47 blockers, the decoy receptor maplirpacept was generated by directly linking the sequences encoding the N-terminal CD47 binding domain of human SIRPα with the Fc domain of human IgG4 (**Figure 1**). The SIRPα region of maplirpacept blocks the CD47 “don’t eat me” signal, while the Fc region binds to Fcγ receptors on macrophages and delivers a moderate pro-phagocytic signal. Wildtype human SIRPα was found to have moderate binding to human CD47 (10) compared to anti-CD47 mAbs (12); we hypothesized that this moderate binding would mitigate binding to RBCs. Maplirpacept is a 77-kDa disulfide-linked, N-glycosylated homodimer consisting of two identical amino acid chains (**Supplementary Figure 1A**).

**Figure 2:**
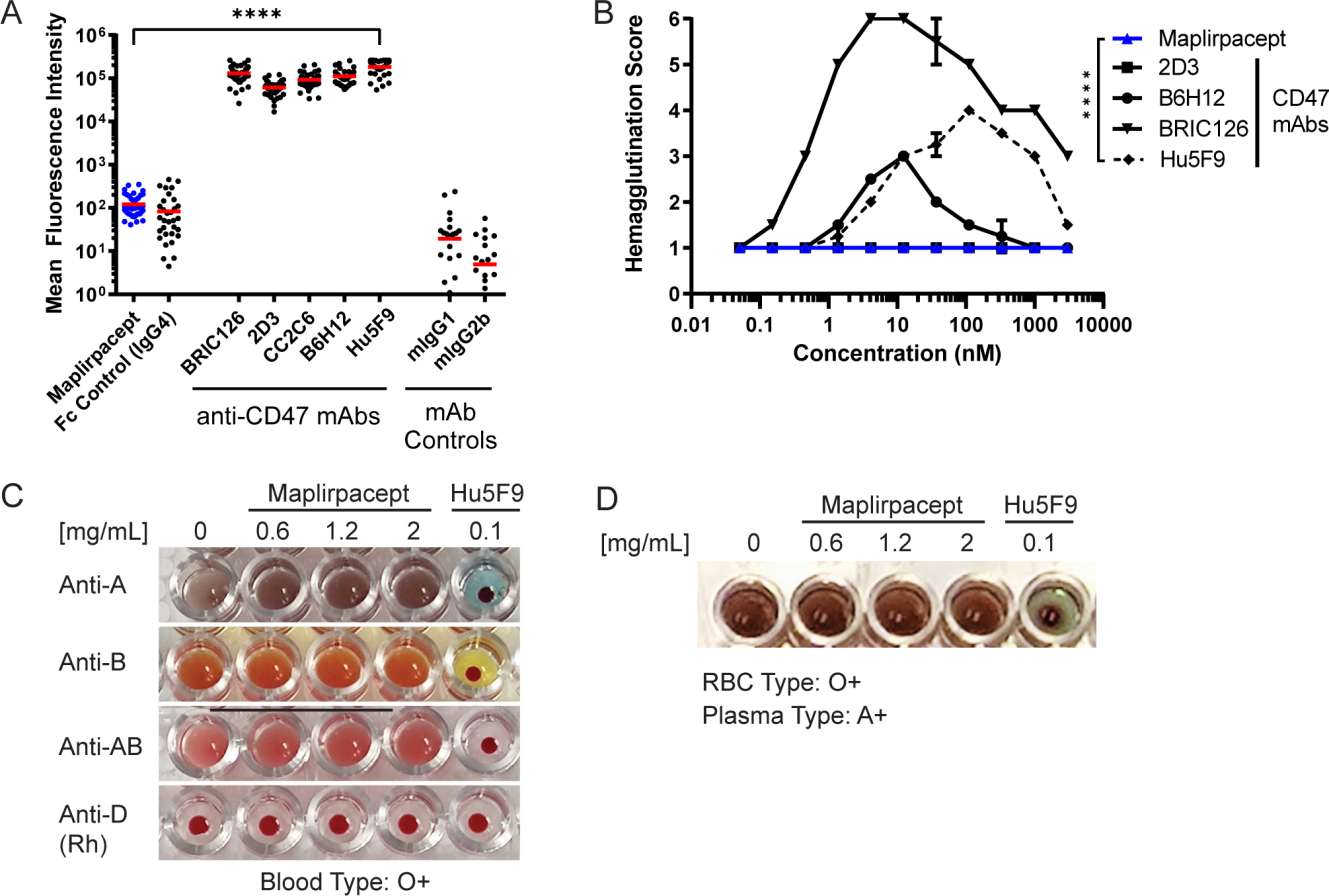
Maplirpacept binds minimally to erythrocytes and does not interfere with blood transfusion compatibility tests. **(A)** Binding of maplirpacept or anti-CD47 mAbs (clones BRIC126, 2D3, CC2C6, B6H12 or Hu5F9) to human RBCs at saturating concentrations and analyzed by flow cytometry; graph represents mean fluorescence intensity (n=43). **(B)** Mean agglutination of human RBCs by maplirpacept and anti-CD47 mAbs. RBCs from healthy donors were treated with maplirpacept or anti-CD47 mAbs overnight. The extent of hemagglutination was assessed by scoring each well on a scale of 1 (absent) to 6 (complete agglutination) (n=2). **(C)** Blood typing of whole blood from healthy donors spiked with indicated concentrations of maplirpacept or anti-CD47 mAb (Hu5F9). **(D)** Indirect Anti-globulin Test with plasma spiked with indicated concentrations of maplirpacept or anti-CD47 mAb (Hu5F9) and whole blood from healthy donors. Error bars represent SEM. Statistical significance was determined by one-way-ANOVA **(A)** or two-way ANOVA**(B)** (****P <0.0001);.

To determine the extent of maplirpacept binding to RBCs relative to other CD47 mAbs, test agents were incubated with human RBCs from 43 donors and protein binding was detected by flow cytometry. Maplirpacept binding to RBCs is not significantly different from Fc control binding which was approximately 3000-fold less than other anti-CD47 antibodies (**Figure 2A**). To determine if these differences in binding also led to differential hemagglutination, maplirpacept and anti-CD47 mAbs were incubated overnight at various concentrations with human RBCs and assessed visually for hemagglutination on a score from 1 to 6 with 1 representing the absence of hemagglutination and 6 representing complete hemagglutination. Maplirpacept led to no discernable hemagglutination at every concentration tested (up to 3 µM). In contrast, the majority of the anti-CD47 Abs induced some level of hemagglutination (**Figure 2B**).

To distinguish whether this limited binding precluded maplirpacept from interference in blood typing tests, RBC suspensions from healthy donors representing all 8 ABO-Rh blood groups were spiked with maplirpacept at 0.6, 1.2 or 2 mg/ml or anti-CD47 (clone Hu5F9). Blood typing was performed and observed for the presence or absence of hemagglutination. The addition of maplirpacept did not cause agglutination of test RBCs and produced the expected blood typing result in a standard forward blood typing test (**Figure 2C**, **Supplementary Table 2A**). No interference was observed at the highest concentration tested. However, addition of an anti-CD47 mAb caused pan-agglutination as well as false positive blood typing results.

Other routine blood testing also includes the indirect antiglobulin test (IAT), which mimics the *in vitro* compatibility crossmatching test performed prior to a blood transfusion. With this test, Hu5F9 gave false positive results while maplirpacept produced results identical to the control serum condition. Additionally, no interference was observed when maplirpacept was added to conditions that produced agglutination in a standard IAT across a range of blood types (**Figure 2D**, **Supplementary Table 2B**).

### Maplirpacept is a potent inducer of phagocytosis *in vitro*

Through flow cytometry, maplirpacept was determined to bind to several cell lines representing hematological tumors (**Supplementary Figure 2A**, mean EC_50_ 361 ± 294nM) and primary cells (**Supplementary Figure 2B**) obtained from blood of B-ALL, T-ALL, MDS and AML patients (mean EC_50_ 517 ± 112 nM).

**Figure 3:**
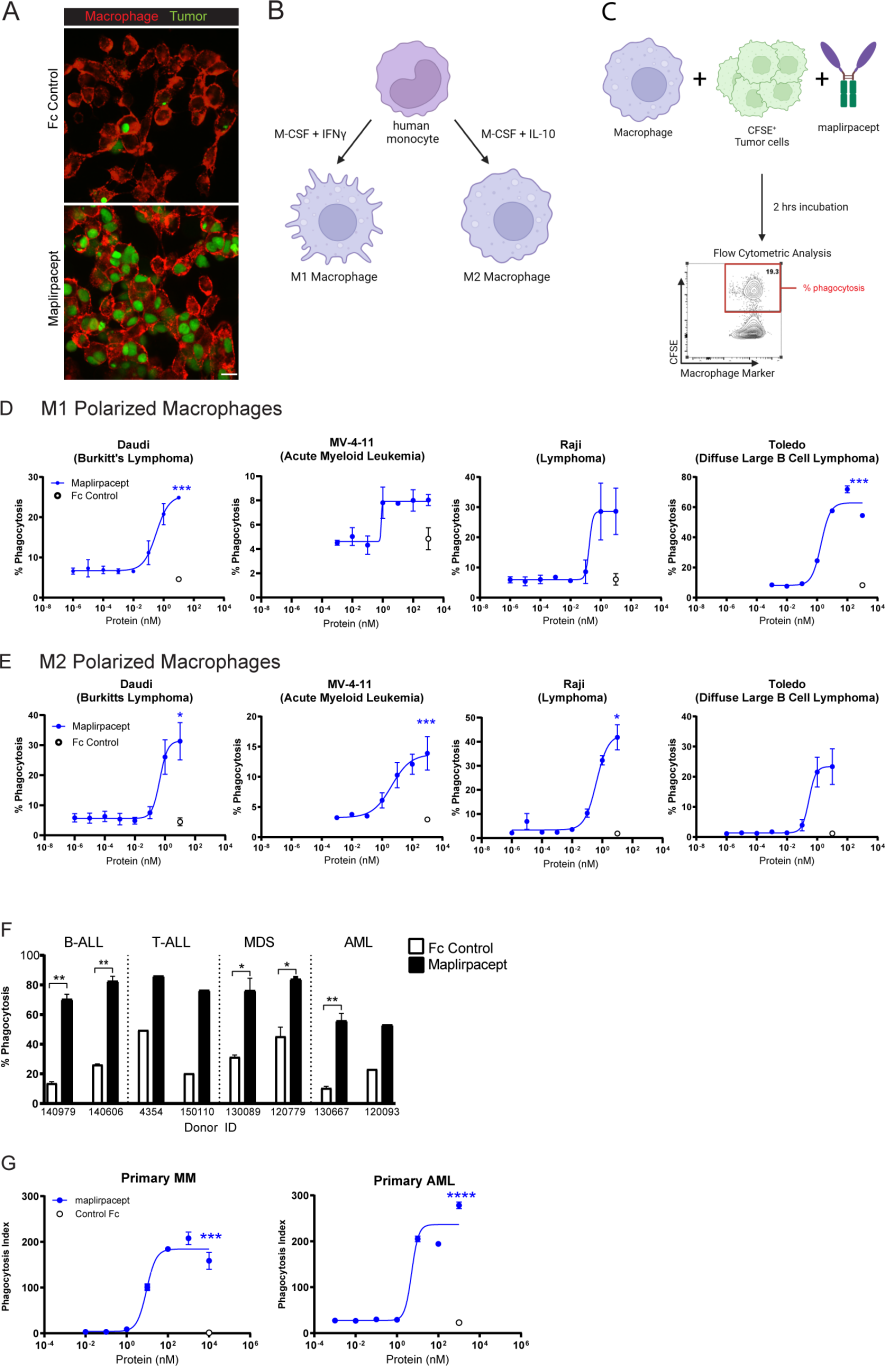
Maplirpacept induces potent phagocytosis of tumor cells *in vitro*. **(A)** Representative scanning confocal microscopy images after macrophages were co-cultured with OCI-AML-2 tumor cells for 2 hours in the presence of 1 µM maplirpacept or control IgG4 Fc protein. Tumor cells and macrophages are stained green and red, respectively. Scale bar 10 µM. **(B)** Schematic for generating M1 and M2 polarized macrophages and **(C)** conducting flow cytometry-based phagocytosis assays. **(D)** M1 or **(E)** M2 polarized macrophage mediated phagocytosis of established human tumor cell lines with titrating concentrations of maplirpacept (blue) or Fc Control (open circles) (n=2) **(F)** Macrophage mediated phagocytosis of B-ALL, T-ALL, MDS and AML primary patient samples in the presence of 1 µM maplirpacept or Fc Control (n=2). **(G)** Macrophage mediated phagocytosis of primary MM and AML patient samples in the presence of titrating concentrations of maplirpacept or 10 µM Fc control via confocal microscopy (n=4). Error bars represent SEM. Statistical significance was determined using unpaired t-test (*P <0.05, **P<0.01, ***P<0.001, ****P<0.0001). Created with BioRender.com

The ability of maplirpacept to induce phagocytosis was observed through confocal microscopy, by co-culturing the AML cell line OCI-AML-2 (green) with M1 polarized macrophages (red) (**Figure 3A**). The addition of maplirpacept to the co-culture induced uptake of tumor cells by macrophages. We also assessed phagocytosis of tumor cells by flow cytomtery by macrophages that were polarized to M1 or M2 with either IFNγ or IL-10 respectively (**Figure 3B**). Polarized macrophages were co-cultured with CSFE stained tumor cells in the presence of titrating concentrations of maplirpacept. The extent of phagocytosis was reported as the percentage of live macrophages that were positive for CFSE stained tumor cells (**Figure 3C**). Increasing concentrations of maplirpacept resulted in an enhanced phagocytic response over baseline with all cell lines tested with both M1 and M2 polarized macrophages (**Figures 3D, E**). At the highest concentration, maplirpacept induced at least a 2-fold increase in phagocytosis over Fc control in all conditions and cell lines. In addition to triggering phagocytosis of human tumor cell lines, maplirpacept also induced phagocytosis of malignant cells from blood of B-ALL, T-ALL, MDS and AML patients (**Figure 3F**). As observed with cell lines described previously, maplirpacept demonstrated a dose-dependent increase in phagocytosis of primary multiple myeloma cells and AML cells (**Figure 3G**). Collectively, these *in vitro* data suggest that maplirpacept induces robust macrophage-mediated phagocytosis of hematological tumor cell lines and patient cells.

As a “don’t eat-me” signal, CD47 is widely expressed on normal non-malignant cells. Thus, whether maplirpacept triggers phagocytosis of non-malignant cells was also determined. Maplirpacept demonstrated less phagocytosis of monocytes and RBCs compared to Hu5F9. Additionally, maplirpacept induced phagocytosis of T cells, B cells and platelets at similar levels to Hu5F9 (**Supplementary Figure 3A**). Moreover, thrombocytopenia was a common treatment emergent adverse event among patients treated with ontorpacept, another SIRPα-Fc decoy receptor (15). To determine if maplirpacept preferentially induces the phagocytosis of tumor cells over platelets, macrophages were cultured with platelets alone, tumor cells alone or platelets and tumor cells together in the presence of maplirpacept or Fc control. Adding tumor cells to platelets reduced platelet phagocytosis, whereas tumor cell phagocytosis was minimally impacted by the presence of platelets (**Supplementary Figures 3B-D**). This suggests that in the presence of maplirpacept, the phagocytosis of malignant cells is favored over platelets.

### Maplirpacept effectively controls tumor burden in a variety of *in vivo* hematological models

**Figure 4:**
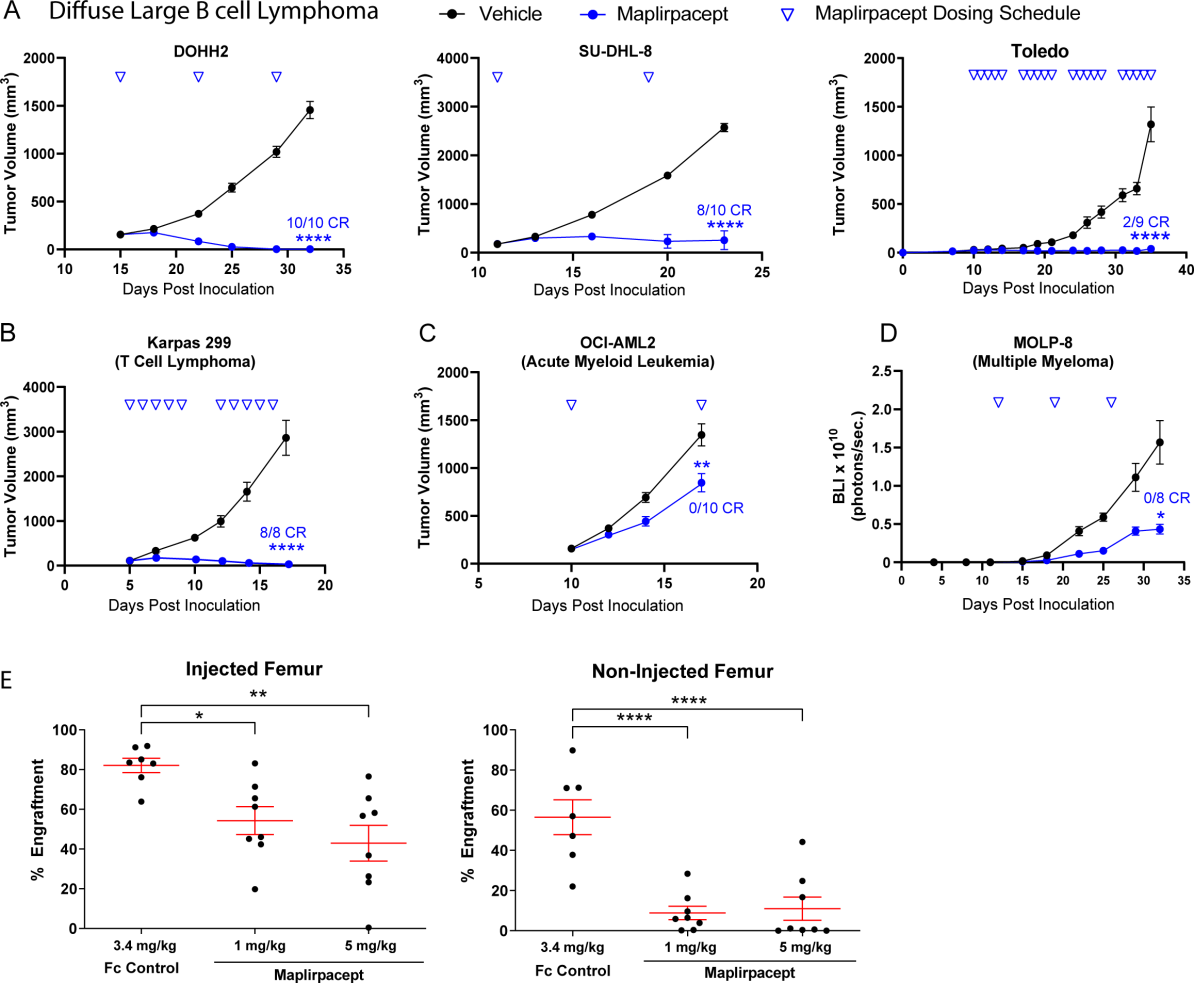
Maplirpacept controls tumor burden in *in vivo* models of hematological malignancy. Tumor growth of **(A)** DLBCL models (DOHH2 [n=10], SUDHL8 [n=10], Toledo [n=9]), **(B)** Karpas 299 (n=8) and **(C)** OCI-AML2 (n=10) implanted subcutaneously in immune deficient mice and dosed with maplirpacept at 3 mg/kg (Karpas 299), 10 mg/kg (DOHH2, Toledo, OCI-AML2) or, 50 mg/kg (SU-DHL-8). Dosing was initiated when initial tumor volumes reached 100-150 mm^3^. Toledo study had 5/9 unmeasurable tumors at the beginning of dosing. **(D)** Tumor growth of luciferase expressing MOLP-8 tumors implanted intravenously and treated with 10 mg/kg maplirpacept subcutaneously. Tumor growth was measured by bioluminescent imaging (BLI), (n=8). **(E)** NOD.SCID mice were preconditioned with sublethal irradiation and anti-CD122 antibody and then transplanted with AML patient derived mononuclear cells by intrafemoral injection. Treatment with maplirpacept (1 or 5 mg/kg i.p. 3×/week for 4 weeks) or equimolar Fc Control was initiated 14 days post-transplantation. The percent AML engraftment (% cells expressing human CD45 and CD33) was assessed by flow cytometry. Each symbol represents one mouse (Fc Control, n =7; Maplirpacept. n=8). Error bars represent SEM. Statistical significance was determined by one-way ANCOVA **(A–D)** or one-way ANOVA **(E)** (*P <0.05, **P<0.01, ****P<0.0001). CR=complete responder (defined as unmeasurable tumor at study end for subcutaneous models (not necessarily TGI endpoint in some studies) and BLI equivalent to baseline in IV models).

To determine if the anti-tumor effects observed *in vitro* are translated *in vivo* with human tumors, immune deficient mice were implanted with various human tumor cells to generate xenograft models. Maplirpacept was effective in reducing tumor burden in multiple subcutaneous tumor models of hematological malignancies (**Figures 4A–D**). Notably, tumors were cleared in DOHH2, SU-DHL-8 and Toledo subcutaneous models of DLBCL (**Figure 4A**) and the Karpas 299 model of T cell Lymphoma (**Figure 4B**). Maplirpacept also induced TGI of 38% in the OCI-AML-2 model of AML (**Figure 4C**). In addition, maplirpacept reduced tumor burden by 72% in the MOLP-8 disseminated model of multiple myeloma, (**Figure 4D**), which is more representative of patient hematological malignancies than subcutaneous xenograph models.

To determine if the effects of maplirpacept observed with cell lines could also be replicated in primary patient derived models, peripheral mononuclear cells collected from an AML patient were injected intrafemorally into NOD.SCID mice and allowed to engraft for 2 weeks before initiating treatment with either maplirpacept or Fc control administered 3x per week for 4 weeks. At the end of treatment, the level of AML engraftment (% cells expressing human CD45 and CD33) was assessed by flow cytometry. Maplirpacept treatment at even the lowest dose resulted in a 34% reduction in mean engraftment in the injected femur and an 84% reduction in the non-injected femur (**Figure 4E**). Collectively, these experiments show that maplirpacept has potent anti-tumor effects in a variety of hematological malignancies *in vivo*.

### Maplirpacept facilitated phagocytosis is further enhanced when combined with other therapeutics

**Figure 5:**
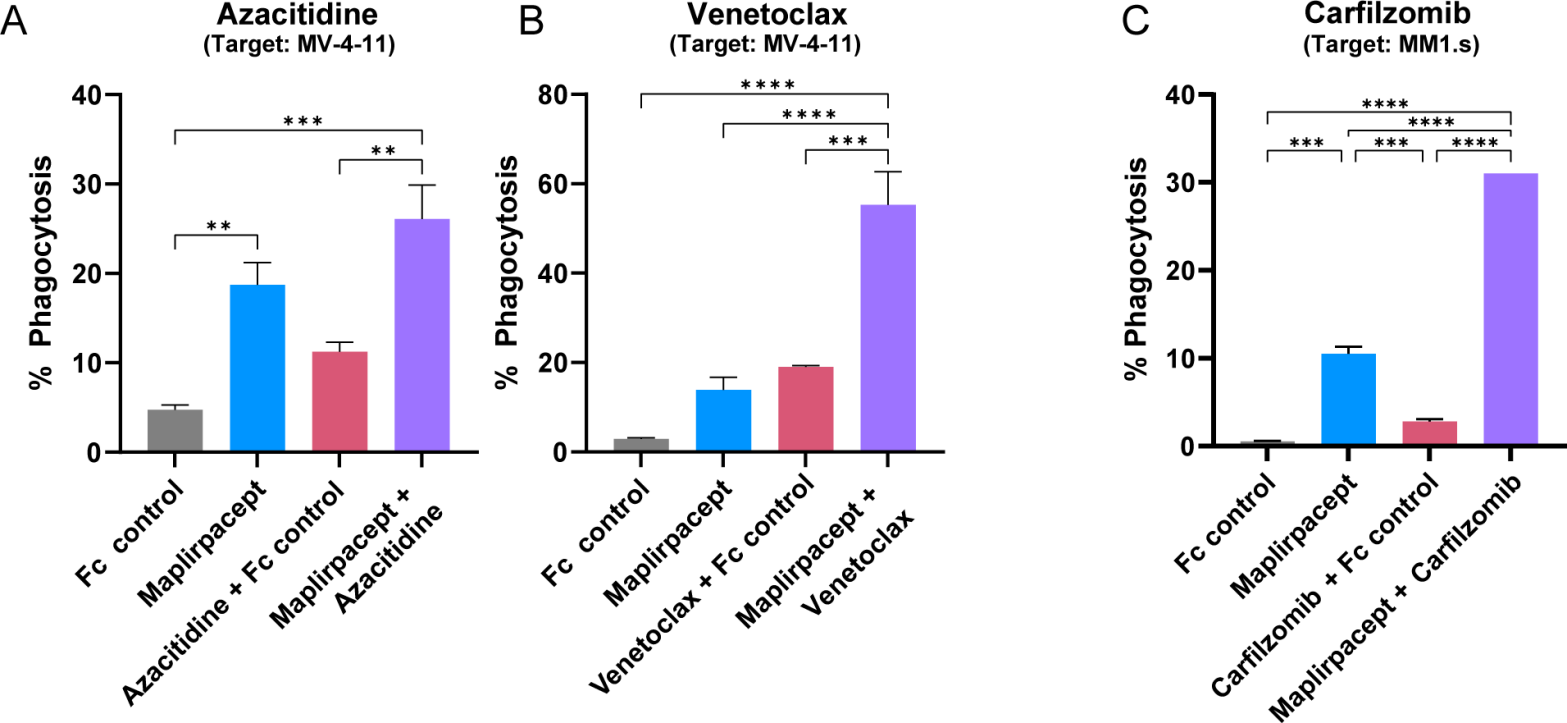
Maplirpacept facilitated phagocytosis is further enhanced in combination with other therapeutics. **(A, B)** Percent phagocytosis of MV-4-11 cells by M2 polarized macrophages. Both macrophages and MV-4-11 cells were treated with either **(A)** 3 µM azacitidine or **(B)** 0.5 µM venetoclax for 72 hrs prior to co-culturing together with 1 µM maplirpacept or 1 µM Fc Control. **(C)** Percent phagocytosis of MM1.s cells treated with 10 nM carfilzomib 48 hrs by M1 polarized macrophages. Treated MM1.s cells and macrophages were co-incubated with 0.1 µM maplirpacept or Fc Control. Data representative of 2 experiments. Error bars represent SEM. Statistical significance was determined by one-way ANOVA (**P<0.01, ***P<0.001, ****P<0.0001).

While maplirpacept alone facilitates robust phagocytosis of different tumor cells, we sought to determine if phagocytosis could be further enhanced by combining maplirpacept with other clinically relevant therapeutic agents such as chemotherapies. As chemotherapy treated cells become apoptotic, they begin to express “eat-me” signals, which could potentially synergize with CD47 blockade (16). Combining maplirpacept with the hypomethylating agent azacitidine or the Bcl-2 inhibitor venetoclax, both of which are used to treat newly diagnosed elderly unfit AML patients ineligible for intensive chemotherapy (17), enhanced the phagocytosis of the AML cell line MV-4-11 compared to either chemotherapy alone (**Figures 5A, B**). The concentrations of azacitidine or venetoclax tested reduced tumor cell viability without affecting macrophage viability (**Supplementary Figures 4A, B**). Similarly, combining maplirpacept with the proteasome inhibitor carfilzomib, a first line treatment of multiple myeloma, increased phagocytosis of the multiple myeloma cell line MM1.s at least 3-fold over maplirpacept or carfilzomib only treatment (**Figure 5C**). The concentrations of carfilzomib that was tested only affected target cells but not macrophage viability (**Supplementary Figure 4C**). Overall, these results demonstrate that the use of the use of maplirpacept in combination with clinically approved chemotherapies can potentially be used to improve phagocytosis of tumor cells *in vitro*.

### Maplirpacept improves tumor control *in vivo* when combined with clinically relevant therapeutics

As the combination of maplirpacept with various other therapies led to an improvement in tumor cell phagocytosis compared to either maplirpacept or combination agent alone *in vitro*, we hypothesized that the combination of therapies would also lead to improved control of tumor burden *in vivo*. Subcutaneous xenograft models of hematologic malignancies were used to test the tumor growth inhibition (TGI) capabilities of selected therapeutics in combination with maplirpacept.

**Figure 6:**
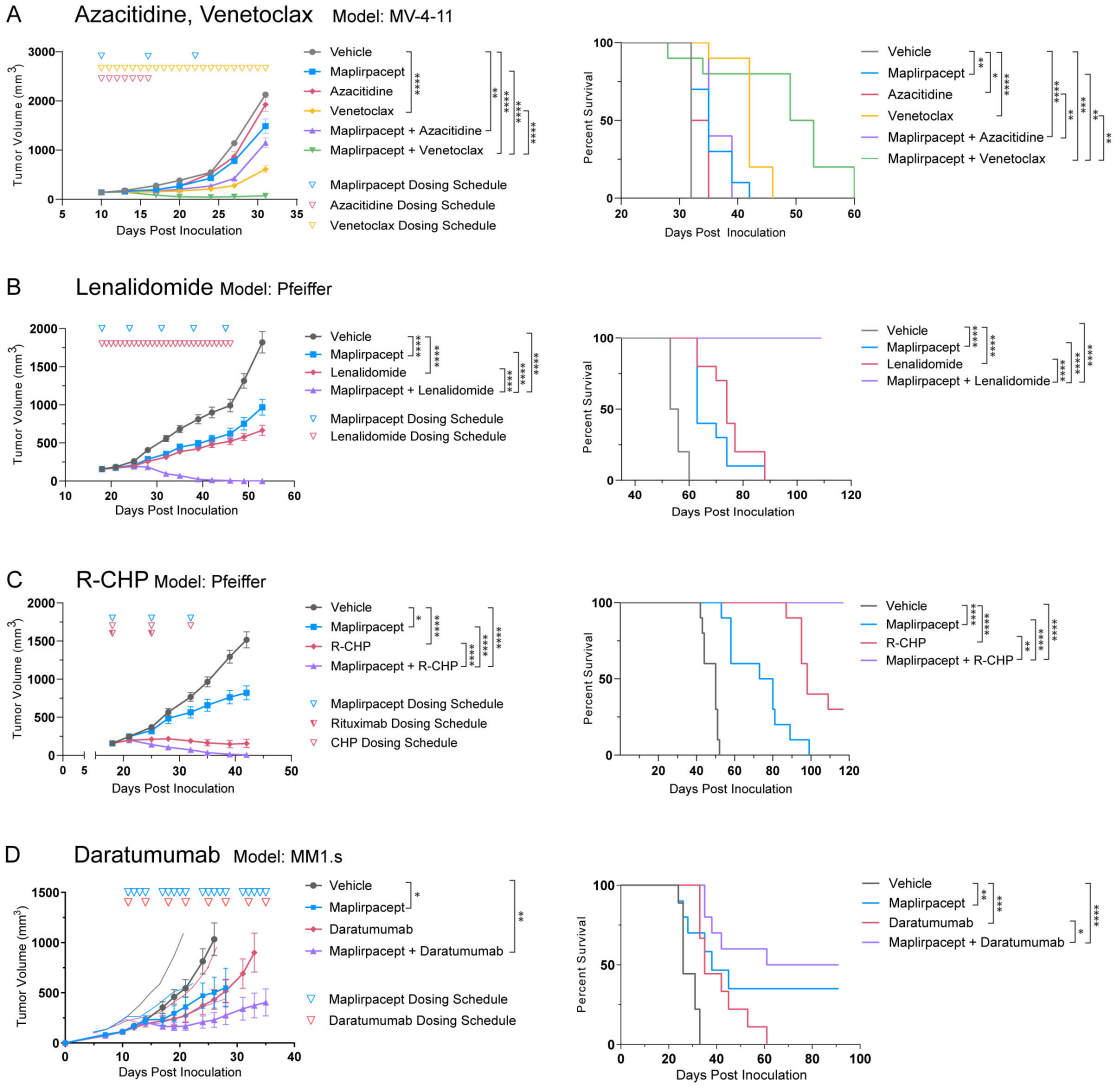
Maplirpacept in combination with various therapeutics can effectively control tumor burden. **(A)** Tumor growth and Kaplan Meier plots of NOD.SCID mice implanted subcutaneously with MV-4-11 and treated with 10 mg/kg maplirpacept SC and/or 3mg/kg azacitidine IP, or 100 mg/kg Venetoclax PO (n=10) **(B)** Tumor growth and Kaplan-Meier plots of NOD.SCID mice implanted subcutaneously with Pfeiffer cells and treated with 30 mg/kg lenalidomide PO and/or 50 mg/kg maplirpacept SC (n=10). **(C)** Tumor growth and Kaplan-Meier plots of NOD.SCID mice implanted subcutaneously with Pfeiffer cells and treated with 20 mg/kg Rituximab (R) IV, 37.5 mg/kg cyclophosphamide **(C)** IV, 2.5 mg/kg doxorubicin (H) IV, 2 mg/kg prednisone (P) PO (n=10), and/or 50 mg/kg maplirpacept SC (n=10). **(D)** Tumor growth and Kaplan-Meier plots of NOD.SCID mice implanted subcutaneously with MM1.s cells and treated with 10 mg/kg daratumumab IP and/or 10 mg/kg maplirpacept IP. (Vehicle, daratumumab, n=9; maplirpacept, maplirpacept + daratumumab, n=10). Tumor volumes are shown as mean ± SEM. Dosing schedule indicated by inverted triangles. Statistical significance was determined by one-way ANCOVA (comparisons with vehicle) or two-way ANCOVA (all other comparisons) for tumor growth plots and log rank mantel-cox test for survival curves. (*P <0.05, **P<0.01, ***P<0.001, ****P<0.0001).

The ability of maplirpacept to enhance the anti-tumor activity of chemotherapy agents azacitidine and venetoclax was assessed using the AML model MV-4-11. Treatment with maplirpacept in combination with azacitidine resulted in a trend towards enhanced inhibition of tumor growth without reaching statistical significance over maplirpacept single agent. Remarkably, combining venetoclax with maplirpacept led to synergistic efficacy compared to either treatment alone (**Figure 6A**).

To further assess the ability of maplirpacept to combine with other chemotherapies, we assessed lenalidomide, an immunomodulatory drug that has emerged as a potential option for patients with DLBCL (18). Treatment with lenalidomide or maplirpacept monotherapy led to moderate control of tumor burden in the DLBCL model Pfeiffer. Remarkably, the combination of the two agents led to a synergistic effect, where all mice in the combination arm were cured of their tumors, leading to significant increases in overall survival (**Figure 6B**). R-CHP (rituximab, cyclophosphamide, doxorubicin, and prednisone) like lenalidomide, has also emerged as a possible treatment option for DLBCL patients. When combined with maplirpacept, R-CHP in the Pfeiffer model also led to synergistic efficacy and full clearance of tumors, which did not occur following treatment with either agent alone (**Figure 6C**).

Another strategy to further improve the efficacy of maplirpacept is to provide additional “eat-me” signals on tumor cells through anti-tumor antibodies. The active Fc domains of these antibodies engage Fcγ receptors on macrophages, which triggers phagocytosis. We thus assessed the ability of maplirpacept to combine with the anti-CD38 antibody, daratumumab, which is currently approved for use in multiple myeloma (19). In the MM1.s model of multiple myeloma, the combination of daratumumab and maplirpacept led to a TGI of 84% (**Figure 6D**). This was an enhancement, although not reaching statistical significance, to the TGI achieved with either maplirpacept (57%) or daratumumab (63%) as a monotherapy. The combination also led to longer survival in comparison to either therapy alone.

Collectively, these data highlight the ability of maplirpacept to add benefit in combination with existing treatment strategies across hematological malignancies, including AML, multiple myeloma, and B cell lymphoma.

## Discussion

The last decade of cancer immunotherapy has seen the prevalence of checkpoint inhibitors targeting the adaptive immune system. However, the importance of the innate immune system in contributing to tumor progression is quickly emerging, especially within the realm of CD47-targeting agents. This is evidenced by the attention devoted to development of CD47 targeting therapies (20). Our findings demonstrate that maplirpacept is an effective CD47 blocker, able to trigger macrophage-mediated phagocytosis of all hematological tumors tested *in vitro* and control tumor burden *in vivo*. Thus, maplirpacept is an attractive emerging therapeutic option for the treatment of hematological malignancies.

A concern with many CD47 blocking therapies is their propensity to cause anemia, as RBCs express high levels of CD47 (21). Unlike anti-CD47 mAbs, minimal binding to human RBCs is observed with maplirpacept, which may be attributed to the restricted mobility of CD47 on the surface of RBCs. Since the affinity of wildtype SIRPα is moderate in comparison to anti-CD47 mAbs, bivalency is required for binding. CD47 molecules are tethered to a tetraspanin network that restricts mobility and precludes clustering (8, 21). SIRPαFc requires pre-clustered CD47 in order to bind to RBCs (10, 22). Indeed, artificial pre-clustering of CD47 with an anti-CD47 antibody with a non-competing epitope of CD47 on human RBCs increased maplirpacept binding (23). The minimal binding of maplirpacept is advantageous as it reduces opsonization of RBCs that would target them for destruction by macrophage mediated phagocytosis causing anemia. Moreover, the limited binding avoids the large antigen sink presented by CD47 expressed on RBCs potentially maximizing engagement with CD47 expressed within tumors. Indeed, maplirpacept has a potentially differentiated safety profile considering the low rates of anemia observed with monotherapy (13).

Maplirpacept is also differentiated in safety in comparison to ontorpacept, another SIRPα Fc fusion protein with an IgG1 Fc region. *In vitro* phagocytosis assays demonstrated that ontorpacept and maplirpacept trigger similar levels of phagocytosis by M1 polarized macrophage whereas maplirpacept triggers less phagocytosis in M2 polarized macrophages compared to ontorpacept (5). Clinically, both agents are dosed differently, with a maximum dose of 2 mg/kg and 24 mg/kg administered for ontopacept and maplirpacept respectively. Thrombocytopenia was the most prevalent related adverse effect of both agents, however maplirpacept led to fewer grade 3 and higher events than ontorpacept despite being administered at higher doses (13, 24). We hypothesize that moderate effector function of IgG4 on maplirpacept might lead to fewer occurrences of thrombocytopenia.

The binding of CD47 blockers to RBCs may not only lead to anemia but can interfere with pre-transfusion blood testing (7). Interference with RBC panel testing has important consequences in transfusion medicine as the presence of irregular antibodies can complicate the selection of suitable RBC units for treated patients. We demonstrated that interference with blood compatibility testing is dependent upon the type of CD47-blocking agent. Maplirpacept, unlike anti-CD47 mAb Hu5F9, does not interfere with blood group serologic testing. This may confer a significant clinical advantage by negating the need for additional approaches to prevent interference.

While maplirpacept is less likely to cause anemia, it was observed to trigger phagocytosis of platelets, which could lead to thrombocytopenia. However, macrophages exhibit preferential engulfment of tumor cells over platelets, and the presence of platelets did not interfere with tumor cell phagocytosis. This specificity for tumors may be attributed to the expression of “eat-me” signals such as calreticulin (25), which can trigger phagocytosis by macrophages, leading tumor cells to be more reliant on expression of “Don’t eat me” signals such as CD47 to avoid being killed compared to healthy cells.

Preclinical testing has shown that maplirpacept is potent as a monotherapy in hematological malignancies, however its therapeutic potential could be further improved in combination with other treatment modalities, including chemotherapies and, monoclonal antibodies. Cytotoxic therapies are known to induce the upregulation of “eat-me” signals like calreticulin on tumor cells, which can trigger phagocytosis. Additionally, cytotoxic therapies induce immunological cell death, which can engage the adaptive immune system in addition to engagement of the innate system triggered by maplirpacept (26). The addition of anti-tumor antibodies to maplirpacept treatment can also improve overall phagocytosis by binding to tumor cells and adding additional “eat-me” signals through the Fc domain.

In this study, the focus was to evaluate combination strategies for treating hematological malignancies, including DLBCL, the most common type of lymphoma, accounting for 30% of all non-Hodgkin’s Lymphoma cases (18). First line standard of care treatment for DLBCL consists of R-CHOP (rituximab, cyclophosphamide, doxorubicin, vincristine, and prednisone) where 20-25% of patients experience relapse after initial treatment (26, 27). Other regimens such as Pola-R-CHP (polatuzumab benl, rituximab, cyclophosphamide, doxorubicin, and prednisone) are approved for use in untreated DLBCL (18). In combination with maplirpacept, R-CHP demonstrated better control of tumor growth and overall survival over monotherapy. Theoretically, each of the five agents within the combination provide non-overlapping methods of action that have the potential to work in concert. For instance, doxorubicin has been shown to modulate macrophage polarization into a more inflammatory state and increase CD47 expression on osteosarcoma cells (28). Doxorubicin-mediated upregulation of CD47 was also observed in triple negative breast cancer (29) This upregulation of CD47 increases the number of maplirpacept molecules that can bind to the tumor cell, thus increasing its likelihood to be phagocytosed by macrophages. Cyclophosphamide, another component of the R-CHP regimen, has been shown to upregulate expression of activating FcγR on murine macrophages (30). This increase would potentiate the phagocytosis triggered by the addition of rituximab and maplirpacept. Indeed, with maplirpacept, R-CHP treatment cleared all Pfeiffer DLBCL tumors in mice.

Combining maplirpacept with the anti-CD38 antibody daratumumab was also efficacious in a model of multiple myeloma. The IgG1 backbone of daratumumab can induce antibody dependent cellular cytotoxicity (ADCC) in addition to antibody dependent cellular phagocytosis (ADCP), therefore adding to the efficacy of maplirpacept (19, 31). Moreover, maplirpacept blocks the inhibitory signaling of SIRPα, which can enhance FcR mediated phagocytosis induced by daratumumab. Studies have shown that activating signals induced by IgG on macrophages must outnumber inhibitory signals by SIRPα 10:1 in order to facilitate ADCP (32). Notably, patients who do not respond to daratumumab were found to have lower baseline levels of CD38 expression prior to treatment compared to patients who had at least a partial response (33). In the case of patients with low CD38 expression, addition of maplirpacept to daratumumab treatment may reduce the threshold for inducing ADCP and overcome resistance to daratumumab.

In this study, we utilized immune-deficient mouse strains to investigate the *in vivo* activity of maplirpacept. SIRPα expressed on NOD murine macrophages can bind human CD47 (albeit with increased affinity than between huSIRPa and huCD47), which allows for the engraftment of human tumors in these strains of mice (10). Despite the increased affinity of NOD SIRPα to huCD47 compared to the human -human interaction, maplirpacept can still reduce tumor burden, suggesting that it successfully blocks the interaction between NOD muSIRPα and huCD47 and still triggers NOD macrophages to phagocytose human tumors. We also tested this binding in the utilized CB17.SCID strain and found that huCD47 was able to bind CB17.SCID derived murine macrophages, albeit to a lesser extent than NOD derived macrophages (data not shown). Conversely, huSIRPα binds with low affinity to NOD muCD47 and therefore the effect of maplirpacept on eliciting anemia in murine models has limited ability to predict anemia in human systems. Additionally, huSIRPα demonstrated dissimilar binding to human and cynomolgus monkey RBCs, suggesting that cynomolgus monkey models also have limited translatability (5).

The mechanism of action of maplirpacept may not only include engagement of macrophages. Macrophages, in addition to removing tumor cells through phagocytosis, can also function as antigen presenting cells (34). Therefore, maplirpacept mediated phagocytosis by macrophages may ultimately lead to priming of CD8^+^ T cells via cross presentation. Indeed, studies have shown that CD47 blocking moieties can trigger a T cell response (35). This suggests that maplirpacept not only can engage the innate arm of the immune system, but also the adaptive, allowing for durable and long-lasting responses. Further studies in immune-competent models are needed to elucidate the effect of maplirpacept on anti-cancer adaptive immunity.

Collectively, this study demonstrates that maplirpacept has limited binding to RBCs, which potentially reduces the risk for hemolytic anemia compared to other CD47 mAbs. This minimal binding also precludes maplirpacept from interfering with routine blood tests. Additionally, maplirpacept enhances macrophage-mediated phagocytosis of a wide variety of hematological tumor cells *in vitro* and has robust anti-tumor effects *in vivo.* Moreover, preclinical evidence demonstrates that *in vitro* phagocytosis and *in vivo* efficacy can be enhanced when maplirpacept is combined with other therapeutic agents, including anti-tumor antibodies or chemotherapeutic agents. Currently, the efficacy of maplirpacept is being explored in several clinical studies (NCT03530683, NCT05896774, NCT05626322, NCT05675449, NCT05896163, NCT05507541).

## Data Availability

The raw data supporting the conclusions of this article will be made available by the authors, without undue reservation.
