# Supplementary material for: Maplirpacept: a CD47 decoy receptor with minimal red blood cell binding and robust anti-tumor efficacy
Source: Front Immunol. 2025 Feb 26;16:1518787. doi: 10.3389/fimmu.2025.1518787 (PMC11897230; doi:10.3389/fimmu.2025.1518787)
Supplement: Supplementary file 1 [file DataSheet1.docx]

Supplementary Material

# Supplementary Tables

## Supplementary Table 1. Treatment dose and regimen for subcutaneous hematological xenograft tumor models


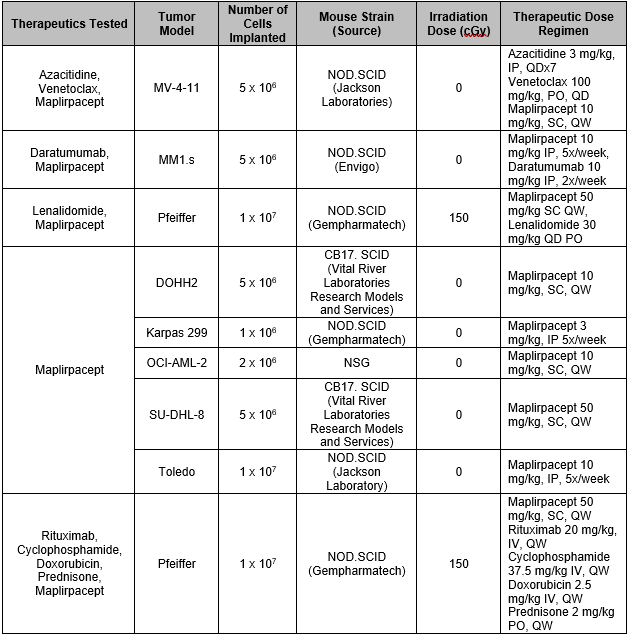


## Supplementary Table 2A:

Maplirpacept Does Not Interfere with Standard Blood Typing Tests. A) Blood typing of whole blood from healthy donors spiked with indicated concentrations of maplirpacept.


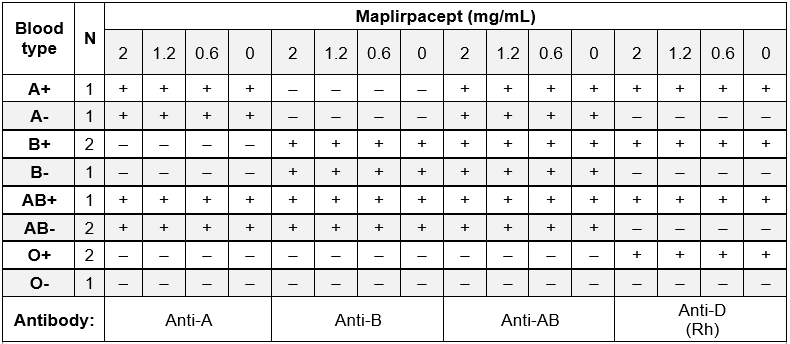


## Supplementary Table 2B:

Anti-CD47 Antibodies Interfere with Standard Blood Typing Tests. B) Indirect Anti-globulin test with plasma (spiked with indicated concentrations of maplirpacept or hu5F9 were incubated with RBCs from healthy donors. Data is represented as presence (+) or absence (-) of agglutination as determined by two independent reviewers blinded to experimental group names. X indicates the conditions that were not tested.


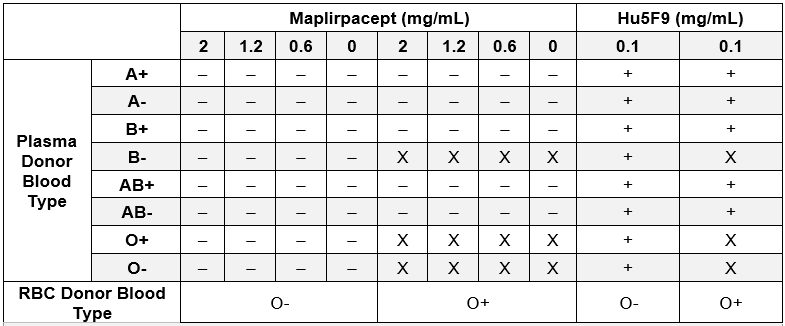


# Supplementary Figures


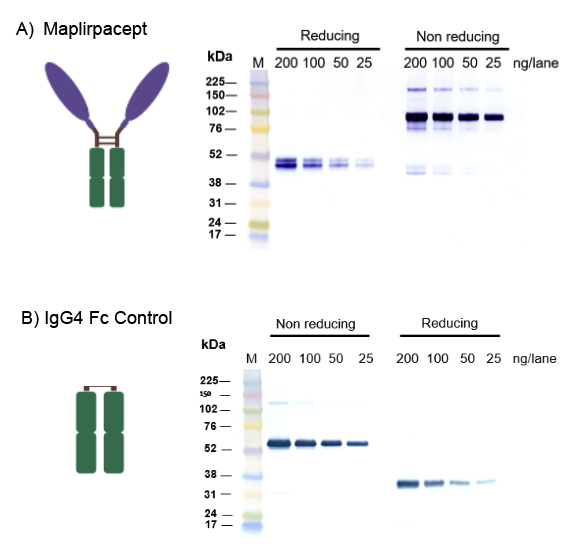


## Supplementary Figure 1. Maplirpacept is a homodimeric SIRPα-hIgG4 Fc fusion protein.

Various concentrations of A) Maplirpacept or B) IgG4 Fc Control were detected via western blot with an anti-hIgGFc antibody under both reducing and non-reducing conditions.


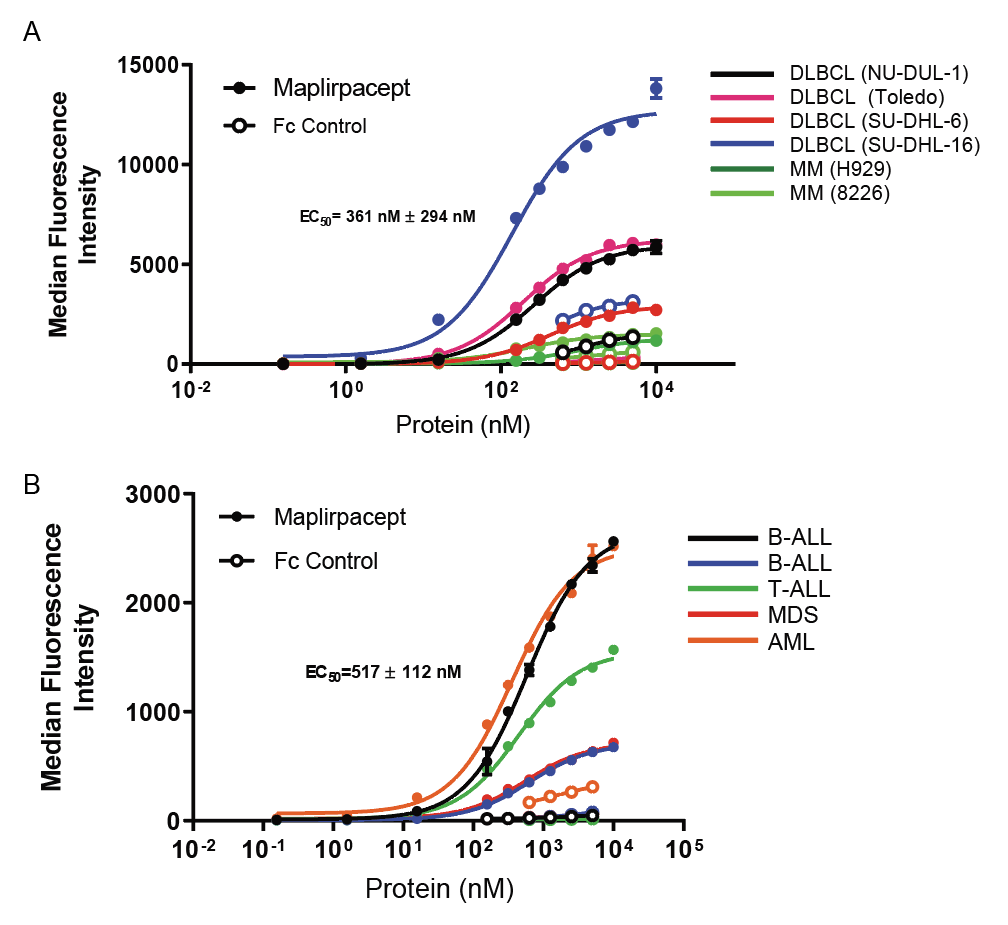


## Supplementary Figure 2. Maplirpacept binds to a variety of tumor cell types.

A) Established human tumor cell lines or B) Primary human tumor samples were incubated with titrated amounts of biotinylated maplirpacept or Fc Control, stained with streptavidin-PE and analyzed via flow cytometry. EC_50_ values are an average of all curves on the graph. Binding curves are generated by least-squares regression.


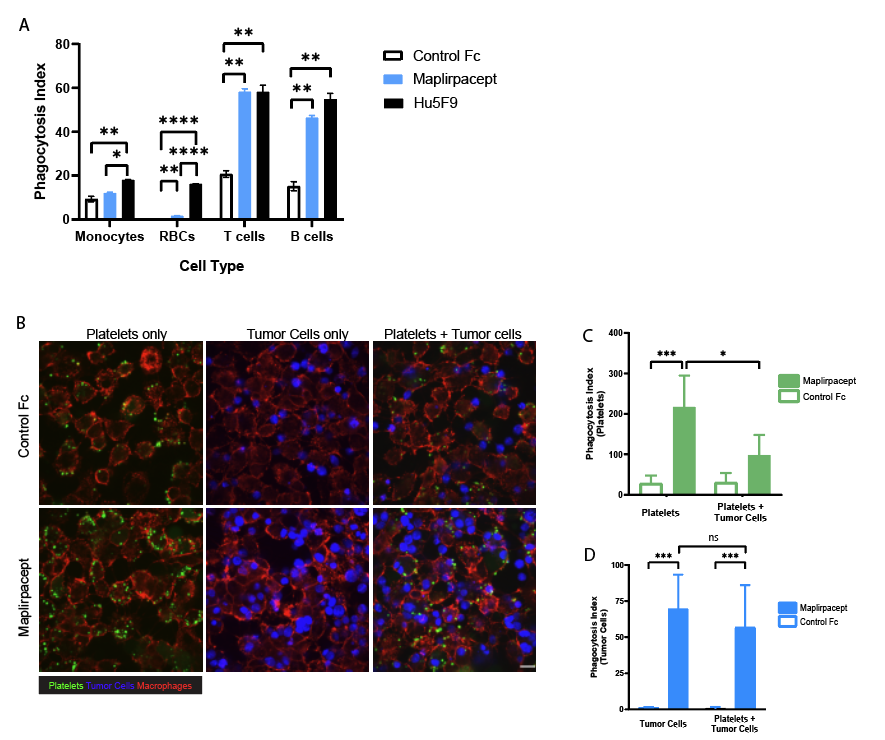


## Supplementary Figure 3. Maplirpacept Induced Phagocytosis of Non-Malignant Cells.

A) Phagocytosis by macrophages of normal monocytes, RBCs, T cells or B cells isolated from human blood in the presence of 1 µM maplirpacept, anti-CD47 Hu5F9 or Control Fc. B) Representative scanning confocal microscopy images after macrophages were co-cultured with tumor cells (SUDHL6) at a 1:5 E:T ratio and/or human platelets (E:T ratio of 1:40) for 2 hours in the presence of 1 µM maplirpacept or Fc control. Tumor cells, platelets and macrophages are stained violet, green and red, respectively. Quantification of C) platelet or D) tumor phagocytosis was performed for experiment described in B. Phagocytosis index is the average number of targets engulfed per macrophage x 100. Data is an average of 3 independent experiments. Error bars represent SEM. Statistical significance was determined by unpaired T-test for (A) and one-way ANOVA for C-D). (*P <0.05, ***P<0.001, ns = not significant).


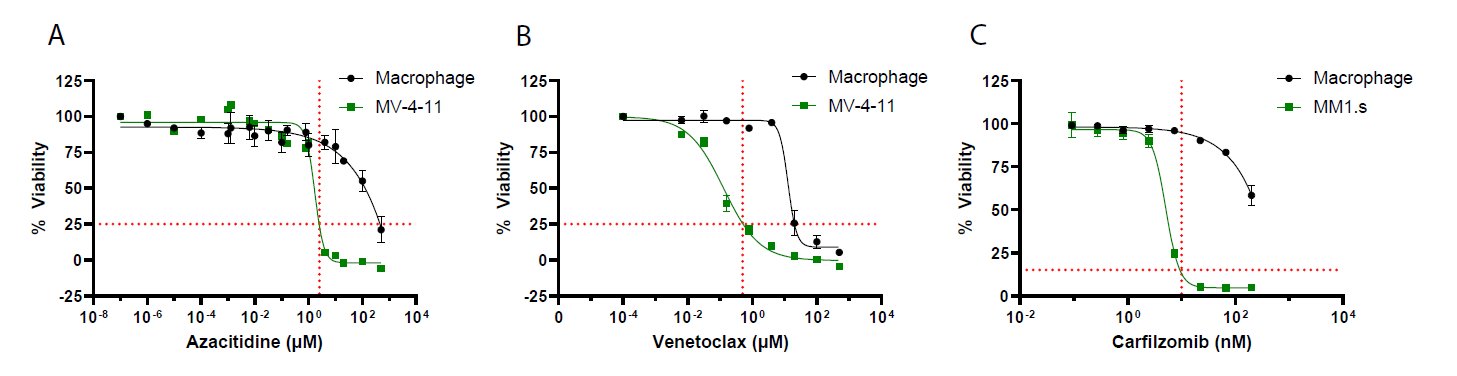


## Supplementary Figure 4. Sensitivity of tumor cells and macrophages to cytotoxic agents.

A-B) MV-4-11 and M2 polarized macrophages were treated with various concentrations of A) Azacitidine or B) Venetoclax for 72 hrs. MM1.s and M1 polarized macrophages were treated with various concentrations of C) Carfilzomib or for 48 hrs. Viability was determined via Cell Titer Fluor assay and is plotted as a mean ± SEM. Red dotted lines indicate concentrations utilized in phagocytosis assays. Data is an average of 2 experiments or 3 technical replicates.
